# Biomarkers predicting clinical outcomes in nasopharyngeal cancer patients receiving immune checkpoint inhibitors: A systematic review and meta-analysis

**DOI:** 10.3389/fimmu.2023.1146898

**Published:** 2023-03-31

**Authors:** Xiaoyan Qian, Haizhu Chen, Yunxia Tao

**Affiliations:** ^1^ Department of Oncology, Henan Provincial People’s Hospital, People’s Hospital of Zhengzhou University, People’s Hospital of Henan University, Zhengzhou, China; ^2^ Breast Tumor Centre, Guangdong Provincial Key Laboratory of Malignant Tumor Epigenetics and Gene Regulation, Department of Medical Oncology, Sun Yat-sen Memorial Hospital, Sun Yat-sen University, Guangzhou, China; ^3^ Phase I Clinical Trial Centre, Guangdong Provincial Key Laboratory of Malignant Tumor Epigenetics and Gene Regulation, Department of Medical Oncology, Sun Yat-sen Memorial Hospital, Sun Yat-sen University, Guangzhou, China; ^4^ Department of Oncology, The Affiliated Hospital of Xuzhou Medical University, Xuzhou, Jiangsu, China

**Keywords:** immune checkpoint inhibitors, nasopharyngeal cancer, biomarker, Epstein-Barr virus, PD-L1 expression, tumor mutation burden, immunotherapy, meta-analysis

## Abstract

**Background:**

Optimal biomarkers to select patients who will benefit most from immunotherapy remain lacking in nasopharyngeal cancer (NPC). This systematic review and meta-analysis aimed to evaluate the association between various biomarkers and clinical outcomes in NPC patients treated with immune checkpoint inhibitors (ICIs).

**Methods:**

Systematic searches of PubMed, Embase, Cochrane Library, and Web of Science databases were performed up to October 2022. Studies evaluating the association between biomarkers and intended outcomes of ICIs were included. The pooled odds ratio (OR) and hazard ratio (HR) with 95% confidence intervals (CIs) were calculated, respectively, for the objective response rate (ORR) and progression-free survival (PFS) under fixed or random-effect models.

**Results:**

A total of 15 studies involving 1,407 patients were included. The pooled analysis indicated that NPC patients with lower plasma Epstein-Barr virus (EBV) DNA level at baseline (OR = 2.14, 95% CI: 1.46-3.14, *P* < 0.001), decreased EBV DNA load during immunotherapy (OR = 4.57, 95% CI: 2.24-9.34, *P* = 0.002) and higher programmed cell death-ligand 1 (PD-L1) expression (OR = 2.35, 95% CI: 1.36-4.09, *P* = 0.002) had superior ORR than the counterparts. No significant differences of ORR were observed between positive PD-L1 expression and negative PD-L1 expression (OR = 1.50, 95% CI: 0.92-2.45, *P* = 0.104), as well as higher tumor mutation burden (TMB) and lower TMB (OR = 1.62, 95% CI: 0.41-6.44, *P* = 0.494). Patients with lower plasma EBV DNA level at baseline obtained a significant benefit on PFS than those with higher plasma EBV DNA level (HR = 0.52, 95% CI: 0.42-0.63, *P* < 0.001). There were no differences in PFS between decreased EBV DNA load and increased EBV DNA load during immunotherapy (HR = 0.51, 95% CI: 0.22-1.17, *P* = 0.109), higher PD-L1 expression and lower PD-L1 expression (HR = 0.65, 95% CI: 0.42-1.01, *P* = 0.054), positive PD-L1 expression and negative PD-L1 expression (HR = 0.90, 95% CI: 0.64-1.26, *P* = 0.531), lower TMB and higher TMB (HR = 0.84, 95% CI: 0.51-1.38, *P* = 0.684).

**Conclusion:**

Lower baseline plasma EBV DNA level, decreased plasma EBV DNA during immunotherapy, and higher PD-L1 expression are reliable biomarkers predicting better response to ICIs treatment. Lower baseline plasma EBV DNA level was also associated with longer PFS. It is warranted to further explore and better illuminate the utility of these biomarkers in future clinical trials and real-world practice.

**Systematic review registration:**

https://www.crd.york.ac.uk/PROSPERO/, identifier CRD42022324434.

## 1 Introduction

Now is an exciting era of development in immune checkpoint inhibitors (ICIs), which have also exhibited encouraging anti-tumor activity for patients with nasopharyngeal cancer (NPC) in recent years (1–4). However, as one of the most common head and neck malignant tumors in Southeast Asia, especially in southern China (5, 6), NPC has no well-established biomarkers for ICIs up to date.

The widely used biomarker, Epstein-Barr virus (EBV), played an important role in the development and progression of NPC (7, 8). However, it is obscure whether plasma EBV DNA level correlates with the anti-tumor activity of ICIs. Some studies showed that lower baseline plasma EBV DNA level was associated with better objective response rate (ORR) and progression-free survival (PFS) compared with the higher EBV DNA level for NPC patients treated with ICIs (3, 9). Other trials, however, did not demonstrate consistent results, in which patients achieved identical clinical benefits regardless of the EBV DNA level (2).

The predictive value of commonly used biomarkers for ICI efficacy, such as programmed cell death-ligand 1 (PD-L1) expression and tumor mutation burden (TMB), is also unclear in NPC. PD-L1 expression was reported to be associated with clinical outcomes in patients with NPC who received chemoradiotherapy, but the utility for ICI efficacy was not well interpreted. Compared with other solid tumors, the level of TMB is relatively lower in NPC (10, 11). Some studies suggested that NPC patients with lower TMB could also achieve clinical benefits with anti-PD-1/PD-L1 therapies as those with higher TMB (9, 12).

So far, there has been no pooled analysis exploring the impact of EBV DNA, PD-L1 expression, and TMB on the clinical outcomes of ICIs for NPC. Herein, we performed a comprehensive systematic review and meta-analysis with recently accumulated evidence to evaluate the association between the three biomarkers and clinical outcomes in NPC patients treated with ICIs.

## 2 Methods

This systematic review and meta-analysis were conducted according to the Preferred Reporting Items for Systematic reviews and Meta-Analyses (PRISMA) guidelines (13) and were registered on the International Prospective Register of Systematic Reviews (PROSPERO) (register ID: CRD42022324434).

### 2.1 Literature search strategy and eligible study selection

Literature search for studies was performed from electronic databases, including PubMed, Embase, Cochrane Library, and Web of Science databases, by two independent investigators (XYQ and YXT) up to October 10, 2022. The Subject headings and main keywords included: (a) “nasopharyngeal carcinoma”, “nasopharyngeal cancer” or “cancer of nasopharynx”; (b) “immune checkpoint inhibitor”, “immunotherapy”, “anti-PD-1” or “anti-PD-L1”. The complete literature search strategy was displayed in **Supplementary Table S1**.

The main criteria for eligibility are as follows: (1) studies in which NPC patients were treated with ICI monotherapy, or ICI combined with chemotherapy/radiotherapy; (2) studies in which the association between plasma EBV DNA level, PD-L1 expression, TMB and clinical outcomes (ORR, PFS) of ICIs was evaluated; (3) studies in which the related data could be extracted directly or calculated indirectly; (5) studies that were written in English. Exclusion criteria are as follows: (1) studies that were reviews, case reports, comments, or letters; (2) studies that were performed on animals or cells; (3) studies that lacked sufficient information. Two investigators (XYQ and YXT) conducted the study search and selection independently. If there was any disagreement, the third investigator (HZC) reassessed the studies.

### 2.2 Data extraction and quality assessment

We extracted the following information from the eligible studies (1) characteristics of studies (first author, publication year, area, type of studies, sample size, follow-up time); (2) characteristics of patients (age, sex, study drugs, biomarkers). (3) clinical outcomes (ORR and PFS), hazard ratios (HRs), and their corresponding 95% confidence intervals (CIs) for PFS. If the HRs and 95% CIs were not provided directly in the study, Engauge Digitizer software (version 11.1) was applied to extract the coordinates of points on the Kaplan-Meier curves. When the results in both univariate and multivariate analyses were available, results from the multivariate analysis were preferred. The cut-off values of plasma EBV DNA levels, PD-L1 expression, and TMB varied across studies. For plasma EBV DNA and TMB, the lower group was identified by the value of lower than the cut-off in each study, otherwise, it was defined as the higher group. When one study reported more than one category by different cut-off values, one of the results was collected. For PD-L1 expression, two comparative models were applied: higher vs. lower and positive vs. negative. The PD-L1 higher and lower category were identified according to the cut-off value in each study: Yang et al. (3), Ma et al. (2), and Park et al. (12) using 10%, Yang et al. (14) using 15%, while Wang et al. (9) using 25%. The PD-L1 positive and negative categories were identified by a cut-off value of 1%. Two investigators (XYQ and YXT) conducted the data extraction independently.

The quality of the studies included was evaluated by Newcastle-Ottawa (NOS) assessment scale criteria, which involved the selection, comparability, and outcomes of the studies (15). The total scores ranged from 0 to 9 points, and the quality criteria were evaluated as follows: poor quality (< 5 points); medium quality (5-7 points); high quality (> 7 points).

### 2.3 Statistical analysis

The predictive value of EBV DNA, PD-L1 expression, and TMB was assessed in NPC patients treated with ICIs. The categorical meta-analysis was performed by comparing lower plasma EBV DNA level with higher EBV DNA level at baseline, decreased plasma EBV DNA load with increased EBV DNA load during ICIs treatment, higher PD-L1 expression in tissue with lower PD-L1 expression, positive PD-L1 expression in tissue with negative PD-L1 expression, and higher TMB in tissue with lower TMB. The impacts of these biomarkers on the clinical outcomes of ICIs were measured by ORR and PFS. Odds ratio (OR) and 95% CI was applied for the pooled analysis of ORR, with HR and 95% CI for PFS.

Cochran’s Q test and Higgins *I* ^2^ statistic were used to evaluate the heterogeneity among studies (16, 17). For the Q test, a *P* value < 0.05 was considered significant heterogeneity. For *I* ^2^ statistics, heterogeneity was assessed as follows: low (*I* ^2^ < 25%), moderate (25% ≤ *I* ^2^ < 50%), and high (*I* ^2^ ≥ 50%). When there was no significant heterogeneity (*P* value of Q test ≥ 0.05 and *I* ^2^ statistic < 50%), a fixed-effect model was performed for the pooled analysis, otherwise, a random-effect model was used. Publication bias was examined by the Funnel plot (18, 19). Sensitivity analysis was conducted by omitting study by study sequentially. Stata version 15.0 was applied to conduct the statistical analyses. A two-sided *P* value < 0.05 was considered a statistically significant difference.

## 3 Results

### 3.1 Systematic search and study selection

**Figure 1:**
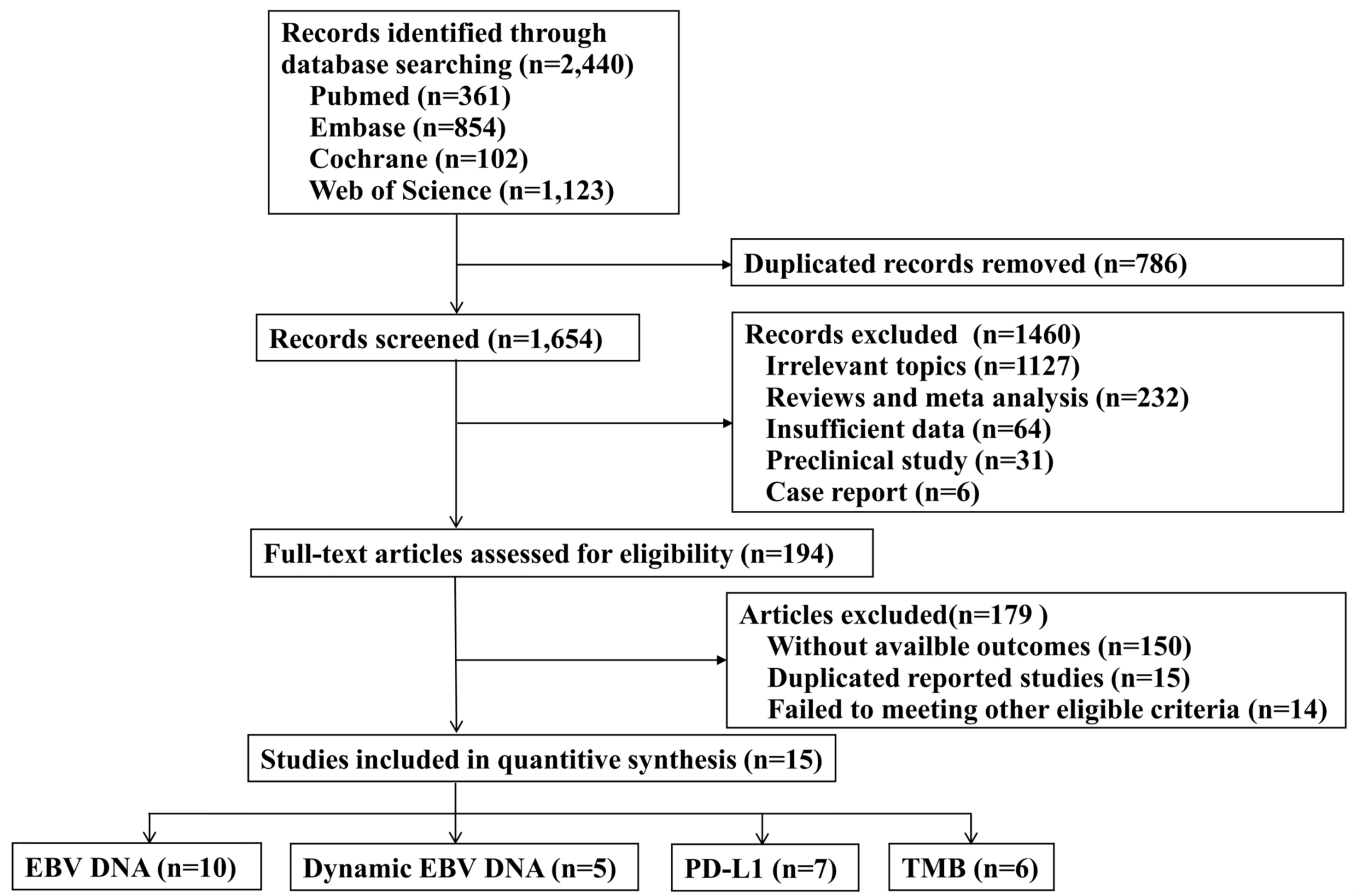
Flow chart of the literature search strategy and eligible study selection process. EBV, Epstein-Barr virus; PD-L1, programmed cell death-ligand 1; TMB, tumor mutation burden.

A total of 2440 records were identified through the electronic databases, with 361 from PubMed, 854 from Embase, 102 from Cochrane, and 1123 from Web of Science. The detailed procedure of literature screening is shown in **Figure 1**. There were 15 relevant studies identified for inclusion in the final analysis (2–4, 9, 12, 14, 20–28), with 13 published articles and 2 conference abstracts, including 1,407 patients.

The quality assessment of the included studies using the Newcastle-Ottawa scale is presented in **Supplementary Table S2**. Two studies were graded as medium quality, with a quality score of 7. Fourteen studies were graded as high quality, with 2 studies scoring 8 and 11 studies scoring 9.

### 3.2 Patients’ characteristics

Of the 15 included studies, 13 studies assessed more than one predictive biomarker. **Table 1** presents the baseline characteristics of the studies included in the systematic review and meta-analysis, including EBV DNA(n=10), dynamic EBV DNA(n=5), PD-L1(n=7), and TMB(n=6). The median age of patients ranged from 44 to 57 years old. The majority of patients were male. All the NPC patients enrolled were recurrence or metastatic diseases. The median follow-up time of the included studies ranged from 5.8 months to 24.7 months.

**Table 1:** Baseline characteristics of the studies included in the systematic review and meta-analysis.

Biomarker	Outcomes	Cut-off value	Study	Region	Type of study	Treatment	Stage	The line of treatment	Sample	Median age (range)	Male (%)	Median follow-up (months)
EBV DNA	ORR	NR	Yang 2021 (14)	China	Prospective (phase II)	Camrelizumab	recurrent or metastatic	>2	156	48 (23–71)	124 (79.5)	14.2 (0.7–27.6)
EBV DNA	PFS	NR	Yang 2021 (3)	China	Prospective (phase III)	Camrelizumab combined with chemo (GP)	recurrent or metastatic	1	134	52 (40–58)	113 (84.0)	10.2 (IQR:7.7–12.7)
EBV DNA	PFS	10,000IU/mL	Xu J 2022 (20)	China	Prospective (phase II)	Toripalimab	recurrent or metastatic	>2	179	46 (22-71)	148 (82.7)	NR
EBV DNA	ORR	10,000IU/mL	Wang 2021 (9)	China	Prospective (phase II)	Toripalimab	recurrent or metastatic	>2	190	46 (22-71)	158 (83.2)	NR
EBV DNA	PFS	1,500 copies/mL	Hua 2021 (21)	China	Prospective (phase II)	Toripalimab combined with radiocherapy	recurrence	1	25	49(IQR: 43.5–52.5)	18 (72.0)	14.6 (IQR: 13.1–16.2)
EBV DNA	ORR	19,000 copies/mL	Even 2021 (22)	France	Prospective (phase II)	Spartalizumab(PDR001)	recurrent or metastatic	>1	82	51 (21–74)	68 (82.9)	NR
EBV DNA	ORR, PFS	30,000 copies/mL	Fang 2018 (23)	China	Prospective (phase I)	Cohort1: Camrelizumab monotherapy	recurrent or metastatic	>1	93	45 (38–52)	75 (81.0)	9.9 (IQR:8.1–11.7)
EBV DNA	ORR, PFS	30,000 copies/mL	Fang 2018 (23)	China	Prospective (phase I)	Cohort2: Camrelizumab combination	recurrent or metastatic	>1	22	44 (34–51)	17 (74.0)	10.2 (IQR:9.7–10.8)
EBV DNA	ORR, PFS	50,000copies/mL	Xu L 2022 (26)	China	Prospective (phase I/II)	Camrelizumab or Nivolumab	recurrent or metastatic	≥1	57	47(25-72)	43(75.4)	5.8
EBV DNA	ORR	1,000 copies/mL	Shi 2022 (4)	China	Prospective (phase II)	KL-A167	recurrent or metastatic	>1	132	49 (26−68)	109 (82.6)	21.7(95%CI: 19.8−22.5)
Dynamic EBV DNA	ORR, PFS	30,000 copies/mL	Fang 2018 (23)	China	Prospective (phase I)	Cohort1: Camrelizumab monotherapy	recurrent or metastatic	>1	93	45 (38–52)	75 (81.0)	9.9 (IQR:8.1–11.7)
Dynamic EBV DNA	ORR	1,000 copies/mL	Shi 2022 (4)	China	Prospective (phase II)	KL-A167	recurrent or metastatic	>1	132	49 (26−68)	109 (82.6)	21.7(95%CI: 19.8−22.5)
Dynamic EBV DNA	ORR	NR	Chiang 2022 (28)	Hong Kong,China	Prospective (phase II)	Bintrafusp alfa	recurrent or metastatic	>1	38	NR	NR	14.9 (1.6-23.3)
Dynamic EBV DNA	PFS	NR	Yang 2021 (3)	China	Prospective (phase III)	Camrelizumab combined with chemo (GP)	recurrent or metastatic	1	134	52 (40–58)	113 (84.0)	10.2 (IQR:7.7–12.7)
Dynamic EBV DNA	PFS	NR	Chen 2022 (27)	China	Prospective (phase II)	Toripalimab combined with chemoradiotherapy	metastatic	≥1	22	54.5 (IQR: 40.5-57.5)	15(68.2)	NR
PD-L1	ORR	1%,10%	Yang 2021 (14)	China	Prospective (phase II)	Camrelizumab	recurrent or metastatic	>2	156	48 (23–71)	124 (79.5)	14.2 (0.7–27.6)
PD-L1	ORR	1%,10%	Ma 2018 (2)	Hong Kong, China	Prospective (phase II)	Nivolumab	recurrent or metastatic	>1	45	57(37-76)	35 (77.8)	12.5 (2.2-22.0)
PD-L1	ORR, PFS	1%, 25%	Wang 2021 (9)	China	Prospective (phase II)	Toripalimab	recurrent or metastatic	>2	190	46(22-71)	158 (83.2)	NR
PD-L1	ORR, PFS	1%,10%	Park 2020 (12)	America	Retrospective	anti-PD-1 antibody therapy	recurrent or metastatic	≥1	42	50 (15–74)	31 (73.8)	13.7 (2.1–55.3)
PD-L1	ORR	1%	Shi 2022 (4)	China	Prospective (phase II)	KL-A167	recurrent or metastatic	>1	132	49 (26−68)	109 (82.6)	21.7(95%CI: 19.8−22.5)
PD-L1	PFS	1%	Hua 2021 (21)	China	Prospective (phase II)	Toripalimab combined with radiocherapy	recurrence	1	25	49(IQR: 43.5–52.5)	18 (72.0)	14.6 (IQR: 13.1–16.2)
PD-L1	PFS	1%, 5%	Mai 2021 (24)	China	Prospective (phase III)	Toripalimab combined with chemo(GP)	recurrent or metastatic	1	130	46(19–72)	124 (85.0)	17.9
TMB	ORR, PFS	2.1muts/Mb	Park 2020 (12)	America	Retrospective	anti-PD-1 antibody therapy	recurrent or metastatic	≥1	42	50 (15–74)	31 (73.8)	13.7 (2.1–55.3)
TMB	ORR, PFS	4muts/Mb	Xu L 2022 (26)	China	Prospective (phase I/II)	Camrelizumab or Nivolumab	recurrent or metastatic	≥1	57	47(25-72)	43(75.4)	5.8
TMB	PFS	2.9muts/Mb	Wang 2021 (9)	China	Prospective (phase II)	Toripalimab	recurrent or metastatic	>2	190	46(22-71)	158 (83.2)	NR
TMB	PFS	NR	Hua 2021 (21)	China	Prospective (phase II)	Toripalimab combined with radiocherapy	recurrence	1	25	49(IQR: 43.5–52.5)	18 (72.0)	14.6 (IQR: 13.1–16.2)
TMB	PFS	NR	Fang 2018 (23)	China	Prospective (phase I)	Cohort1: Camrelizumab monotherapy	recurrent or metastatic	>1	93	45 (38–52)	75 (81.0)	9.9 (IQR:8.1–11.7)
TMB	PFS	NR	Ma 2021 (25)	China	Prospective (phase I)	Camrelizumab or Nivolumab	recurrent or metastatic	>1	60	46 (23–73)	95 (76.6)	24.7 (95%CI:23.3- 26.6)

EBV, Epstein-Barr virus; PD-L1, programmed cell death-ligand 1; TMB, tumor mutation burden; ORR, objective response rate; PFS, progression-free survival; GP, gemcitabine and cisplatin; NR, not reported; IQR, interquartile range; CI, confidence interval.

### 3.3 Pooled analysis of ORR

**Figure 2:**
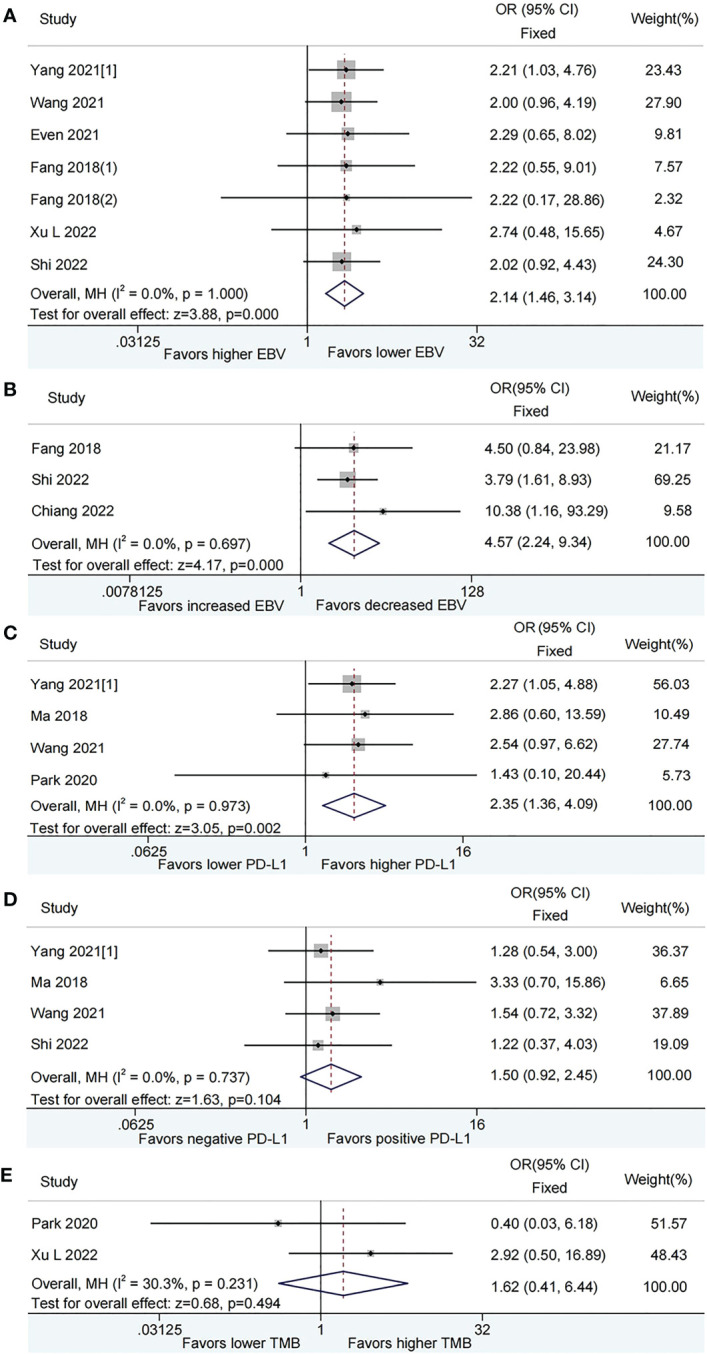
Meta-analysis of the association between biomarkers and objective response rate (ORR). **(A)** baseline plasma Epstein-Barr virus (EBV) DNA level and ORR; **(B)** Dynamic plasma EBV DNA load during immunotherapy and ORR; **(C)** programmed cell death-ligand 1 (PD-L1) expression [higher vs. lower] and ORR; **(D)** PD-L1 expression [positive vs. negative] and ORR; **(E)** tumor mutation burden (TMB) and ORR.

After pooled analysis, patients with lower plasma EBV DNA level at baseline had superior ORR than those with higher plasma EBV DNA level (OR = 2.14, 95%CI: 1.46-3.14, *P* < 0.001, **Figure 2A**). Compared with patients harboring increased plasma EBV DNA load during immunotherapy, those with decreased EBV DNA load obtained a significant benefit on ORR (OR = 4.57, 95%CI: 2.24-9.34, *P* < 0.001, **Figure 2B**). There was no heterogeneity among the studies included.

In the pooled analysis, higher PD-L1 expression was associated with increased ORR than lower PD-L1 expression (OR = 2.35, 95%CI: 1.36-4.09, *P* = 0.002, **Figure 2C**). Nevertheless, there was no significant difference between positive PD-L1 expression and negative PD-L1 expression as for ORR (OR = 1.50, 95%CI: 0.92-2.45, *P* = 0.104, **Figure 2D**). No evidence of heterogeneity was observed among the analysis.

The pooled OR for ORR was 1.62 (95% CI: 0.41–6.44, *P* = 0.494), which indicated that patients with lower TMB had a comparable ORR with those with higher TMB. A moderate level of heterogeneity (*I* ^2^ = 30.3%, *P* = 0.231, **Figure 2E**) was observed among the studies included.

### 3.4 Pooled analysis of PFS

**Figure 3:**
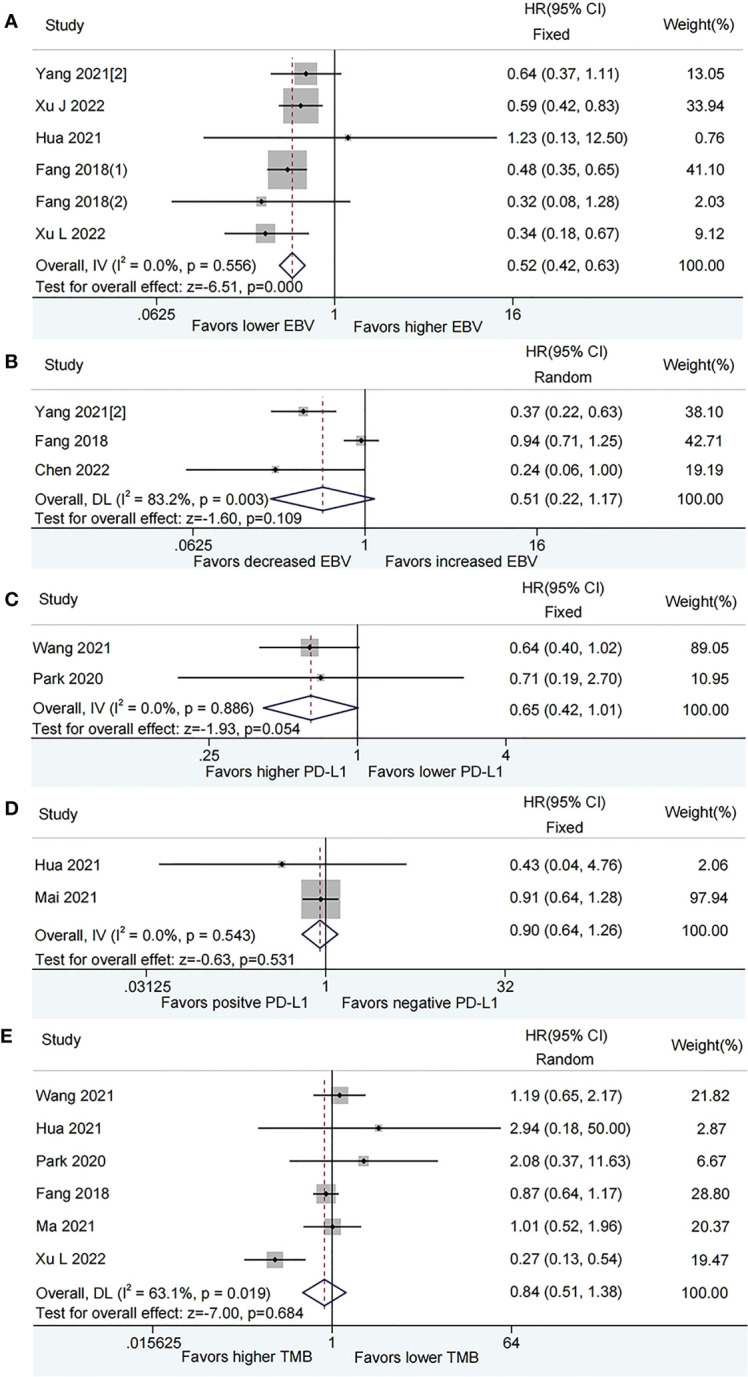
Meta-analysis of the association between biomarkers and progression-free survival (PFS). **(A)** baseline plasma Epstein-Barr virus (EBV) DNA level and PFS; **(B)** Dynamic plasma EBV DNA load during immunotherapy and PFS; **(C)** programmed cell death-ligand 1 (PD-L1) expression [higher vs. lower] and PFS; **(D)** PD-L1 expression [positive vs. negative] and PFS; **(E)** tumor mutation burden (TMB) and PFS.

According to the fixed effects model, patients with lower plasma EBV DNA level at baseline had longer PFS (HR = 0.52, 95% CI: 0.42–0.63, *P* < 0.001, **Figure 3A**) than those with higher plasma EBV DNA level. Patients with decreased plasma EBV DNA load during immunotherapy did not show a significant benefit on PFS than those with increased plasma EBV DNA load (HR=0.51, 95% CI:0.22–1.17, *P*=0.109; **Figure 3B**) by the random-effect model.

The pooled analysis showed that patients with higher PD-L1 expression had a tendency towards longer PFS than those with lower PD-L1 expression, while this did not reach a statistical difference (HR = 0.65, 95% CI: 0.42-1.01, *P* = 0.054, **Figure 3C**), There was no difference in PFS between positive PD-L1 expression and negative PD-L1 expression (HR = 0.90, 95% CI: 0.64-1.26, *P* = 0.531, **Figure 3D**). No evidence of heterogeneity was observed among the analysis.

The forest map did not show that patients with higher TMB have a lower risk of disease progression than those with lower TMB (HR = 0.84, 95% CI: 0.51-1.38, *P* = 0.484, **Figure 3E**) based on a random-effect model.

### 3.5 Sensitivity analysis

The sensitivity analysis, which was conducted by removing one study at each time, showed that the pooled results were not significantly influenced by any single study (**Supplementary Figures S1**, **S2**). Considering the relatively limited number of included studies for PFS of PD-L1 expression and ORR of TMB, sensitivity analysis was not applied to test the potential heterogeneity.

### 3.6 Publication bias

**Figure 4:**
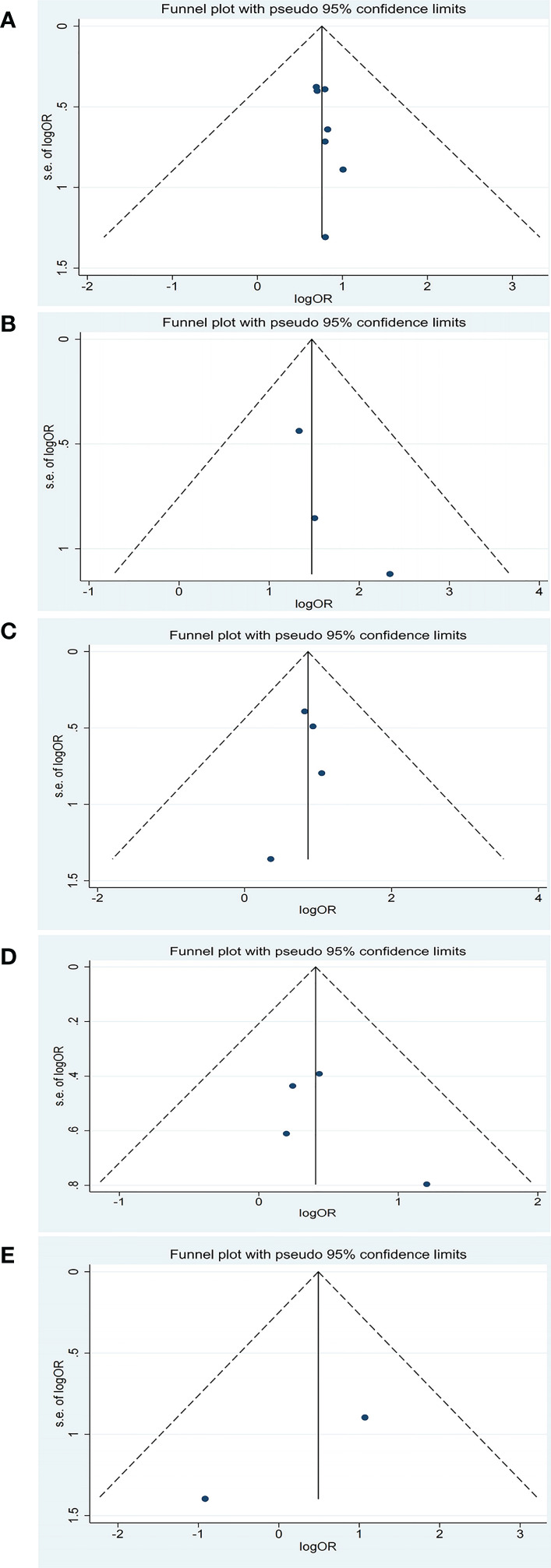
Funnel plot of objective response rate (ORR) for studies reporting biomarkers. **(A)** baseline plasma Epstein-Barr virus (EBV) DNA level; **(B)** dynamic plasma EBV DNA load during immunotherapy; **(C)** programmed cell death-ligand 1 (PD-L1) expression (higher vs. lower); **(D)** PD-L1 expression (positive vs. negative); **(E)** tumor mutation burden (TMB).

**Figure 5:**
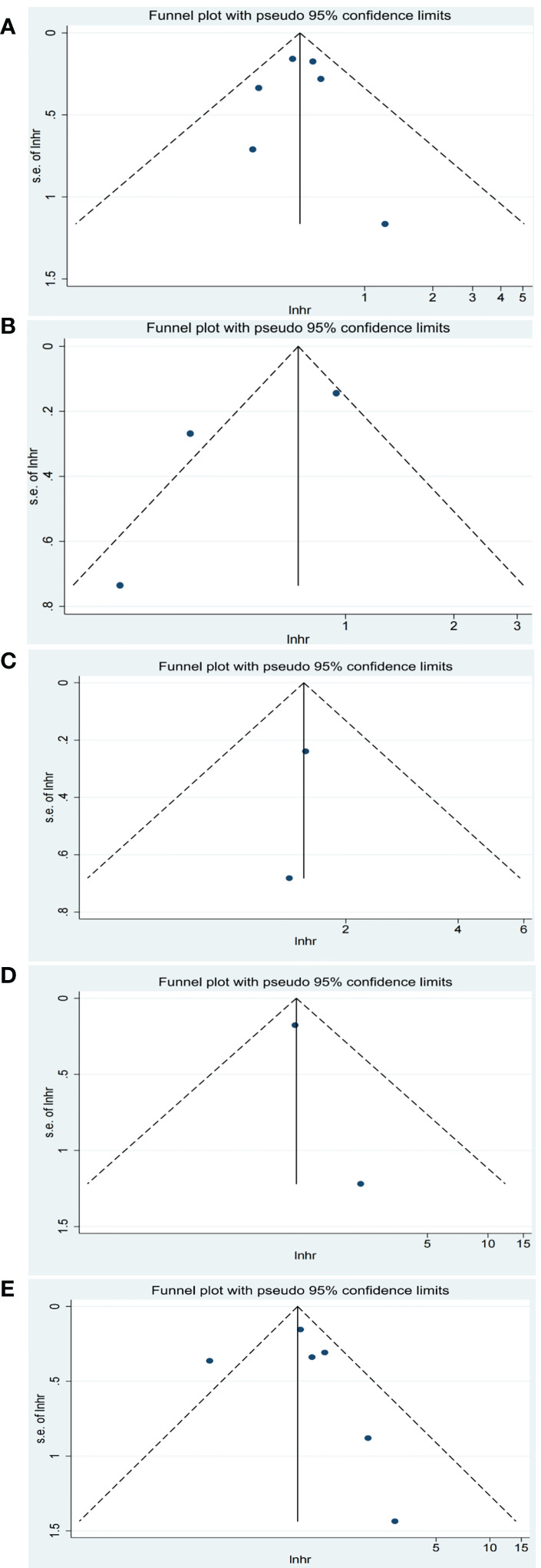
Funnel plot of progression-free survival (PFS) for studies reporting biomarkers. **(A)** baseline plasma EBV DNA level; **(B)** dynamic plasma EBV DNA load during immunotherapy; **(C)** programmed cell death-ligand 1 (PD-L1) expression (higher vs. lower); **(D)** PD-L1 expression (positive vs. negative); **(E)** tumor mutation burden (TMB).

There was a slight asymmetrical according to the funnel plot for PFS of TMB. There was no obvious publication bias for the other pooled analysis when tested by funnel plot (**Figures 4**, **5**).

## 4 Discussion

Though immunotherapy has become an increasingly attractive approach for patients with NPC, the optimal biomarkers to select patients who will benefit most from ICIs remain lacking. To our best knowledge, this meta-analysis is the first and the most comprehensive one that focused on the biomarkers predicting the clinical outcomes of patients with NPC receiving ICIs. In this study, we analyzed the association between plasma EBV DNA level at baseline, dynamic change of plasma EBV DNA level during immunotherapy, PD-L1 expression, TMB, and intended outcomes (ORR and PFS) of ICIs in NPC.

The role of plasma EBV DNA as a clinically useful biomarker in the detection, guiding chemotherapy and radiotherapy, surveillance, and prognostication for NPC has been well established (8, 29, 30). However, it is controversial whether the plasma EBV DNA level was associated with the clinical outcomes of ICIs. Notably, our study observed that NPC patients with lower plasma EBV DNA level at baseline had higher ORR and longer median PFS compared with patients with higher EBV DNA level. In addition, post-treatment EBV DNA decrease was correlated with a better response to ICIs in NPC. One possible underlying mechanism for the pretreatment and the dynamic change of plasma EBV DNA level as a potential indicator for clinical outcomes of NPC patients receiving ICIs might be the tumor evasion from the immune system. The EBV encoding latent membrane proteins and noncoding RNA molecules, limit the actions of interferon and block antigen presentation, which allows NPC cells to escape immune recognition and avoid immune (31, 32). As a result, a heavy load at baseline or an increase post-treatment of plasma EBV DNA level could be correlated with a higher number of NPC tumor cells escaping immune recognition, thus resulting in poor outcomes for patients treated with ICIs (33). Taken together, plasma EBV DNA may pave a way towards the precision immunotherapy approach in NPC. More studies investigating the biological mechanisms underlying those associations are worthwhile to be conducted in the near future.

The predictive value of PD-L1 expression, the most extensively studied biomarker for immunotherapy, though proved to be a useful biomarker in predicting the efficacy of ICIs in lung cancer, esophageal cancer, and other solid carcinomas (34, 35), was still inconclusive in NPC. In our study, no difference was observed with respect to ORR and PFS between positive and negative PD-L1 expression (a cutoff of 1%) in NPC patients receiving ICIs. However, when using a higher cut-off value, a better ORR was observed in high PD-L1 expression. These results manifest that PD-L1 expression has certain predictive utility in NPC, and further considerable studies are warranted to explore the optimal cut-off value of PD-L1 expression to better illuminate the association between PD-L1 expression and outcomes of ICIs.

TMB was emerging as a potential biomarker for immunotherapy in recent decades. Previous studies suggested that higher TMB was associated with a higher number of tumor-neoantigens presented on major histocompatibility complex class (MHC) molecules, which facilitated immune recognition and the response to anti-tumor immunotherapy (36). Our study found that there was no significant correlation between TMB and clinical outcomes in NPC patients receiving ICIs. This may be due to the variable cut-off values of TMB across studies and the distinct tumor microenvironment of NPC from other solid tumors. The relationship between TMB and response to ICIs remains challenging in NPC.

Notably, additional cohort studies explored the association between other biomarkers (eg, human leukocyte antigen [HLA], MHC and the effect on ICIs. In the CAPTAIN trial, a high MHC-II+ cell density in the stroma was found to be associated with improved disease control rate (DCR), longer median PFS, and OS (14). In an international and multicenter study of nivolumab (NCI-9742), they observed that loss of HLA-A and HLA-B was associated with better survival than patients with HLA-A– and HLA-B–intact tumors (2). However, relevant studies were limited, and there was relatively inadequate power to conduct a meta-analysis. Substantial efforts are needed to elucidate the role of these biomarkers in predicting response and prognosis for NPC patients receiving ICIs.

Besides, the definition of biomarkers has been expanded greatly with the evolution of bioinformatics. A combination of ICI prediction methods with tumor prognostic markers at the molecular level has been well applied in multiple carcinomas (37–41). Chi and colleagues established a multi-biomarker prognostic model based on natural killer cell-associated genes in head and neck squamous cell carcinoma (HNSCC) (37). Chen et al. assessed tumor microenvironment (TME) through virtual microdissection of gene expression profiles, classifying the TME of NPC into three immune subtypes to predict immunotherapy responses and prognosis (42). Undoubtedly, these approaches provide new perspectives for evaluating the response and prognosis of immunotherapy. Biomarkers of EBV DNA, PD-L1, and TMB in this study have their advantages. First, they are affordable in price. Secondly, the detection technology is mature and easy to be widely used in clinical. Third, the detection of plasma EBV DNA was non-invasive and can be monitored dynamically.

Several limitations should be considered in this meta-analysis. First of all, the number of studies included in each biomarker for each outcome was relatively small. Only two studies were included in the pooled analysis for PFS of PD-L1 expression and ORR of TMB, and the relatively limited number of included studies may limit the power of analysis. Secondly, the majority of the studies included were from China, which may lead to some inevitable sources of bias. However, this may be due to the fact that the endemic regions of NPC are extremely unbalanced, with 72.8% of new cases in Southeast Asia. The age-standardized rate was 3.0 per 100,000 in China, while 0.4 per 100,000 in white populations (5, 6). The essential reason for publication bias may be the incentives that researchers are more likely to report statistically significant results to be accepted for publication and publishers are more likely to publish studies with statistically significant findings. Thirdly, though overall survival (OS) is also an important outcome to be investigated, the studies reporting the effect of biomarkers on OS were limited to conducte a pooled analysis.

## 5 Conclusion

In conclusion, lower baseline plasma EBV DNA level, decreased EBV DNA load during immunotherapy, and higher PD-L1 expression are reliable biomarkers predicting better response to ICIs treatment. Lower baseline plasma EBV DNA level was also associated with longer PFS. It is warranted to further explore and better illuminate the utility of these biomarkers in future clinical trials and real-world practice.

## Data availability statement

The original contributions presented in the study are included in the article/**Supplementary Material**. Further inquiries can be directed to the corresponding author.

## Author contributions

XQ, HC and YT designed the study, performed the systematic search and selected eligible studies. XQ and HC analyzed the data. XQ wrote the manuscript. HC and YT revised the manuscript. All authors contributed to the article and approved the submitted version.
