# Supplementary material for: Biomarkers predicting clinical outcomes in nasopharyngeal cancer patients receiving immune checkpoint inhibitors: A systematic review and meta-analysis
Source: Front Immunol. 2023 Mar 31;14:1146898. doi: 10.3389/fimmu.2023.1146898 (PMC10102485; doi:10.3389/fimmu.2023.1146898)
Supplement: Supplementary file 1 [file DataSheet_1.docx]

**Supplementary Materials**

**Biomarkers predicting clinical outcomes in nasopharyngeal cancer patients receiving immune checkpoint inhibitors: a systematic review and meta-analysis**

**Contents**

Table S1 Search strategies.........................................................................................................................2

Table S2 Quality assessment of included studies using Newcastle-Ottawa quality assessment scale

............................................................................................................................................ ......................6Figure S1 Sensitivity analysis of studies included in meta-analysis of objective response rate (ORR) for biomarkers..................................................................................................................................................7

Figure S2 Sensitivity analysis of studies included in meta-analysis of progression-free survival (PFS) for biomarkers..................................................................................................................................................8

References………………………….…………………………………………………………………….9

# Table S1 Search strategies

| **Database** | **Search terms** | **Results** |
| --- | --- | --- |
| **PubMed** | #1: "Nasopharyngeal Neoplasms"[Mesh] | 18,578 |
|  | #2:((((((((((((((((Nasopharyngeal Neoplasm[Title/Abstract]) OR (Neoplasm, Nasopharyngeal[Title/Abstract])) OR (Neoplasms, Nasopharyngeal[Title/Abstract])) OR (Nasopharynx Neoplasms[Title/Abstract])) OR (Nasopharynx Neoplasm[Title/Abstract])) OR (Neoplasm, Nasopharynx[Title/Abstract])) OR (Neoplasms, Nasopharynx[Title/Abstract])) OR (Cancer of Nasopharynx[Title/Abstract])) OR (Nasopharynx Cancers[Title/Abstract])) OR (Nasopharyngeal Cancer[Title/Abstract])) OR (Cancer, Nasopharyngeal[Title/Abstract])) OR (Cancers, Nasopharyngeal[Title/Abstract])) OR (Nasopharyngeal Cancers[Title/Abstract])) OR (Nasopharynx Cancer[Title/Abstract])) OR (Cancer, Nasopharynx[Title/Abstract])) OR (Cancers, Nasopharynx[Title/Abstract])) OR (Cancer of the Nasopharynx[Title/Abstract]) | 20,779 |
|  | #3: #1 OR #2 | 23,494 |
|  | #4:((((((((((((((((((((((((((((((((((((((((((((((((((((PD-1[Title/Abstract]) OR (PD-L1[Title/Abstract])) OR (anti-PD-1[Title/Abstract])) OR (anti-PD-L1[Title/Abstract])) OR (immune checkpoint inhibitor[Title/Abstract])) OR (immune checkpoint inhibitors[Title/Abstract])) OR (ICI[Title/Abstract])) OR (ICIs[Title/Abstract])) OR (immune checkpoint blocker[Title/Abstract])) OR (immune checkpoint blockers[Title/Abstract])) OR (ICB[Title/Abstract])) OR (ICBs[Title/Abstract])) OR (immunotherapy[Title/Abstract])) OR (CTLA-4[Title/Abstract])) OR (Anti-CTLA-4[Title/Abstract])) OR (nivolumab[Title/Abstract])) OR (Opdivo[Title/Abstract])) OR (ONO-4538[Title/Abstract])) OR (MDX-1106[Title/Abstract])) OR (BMS-936558[Title/Abstract])) OR (pembrolizumab[Title/Abstract])) OR (SCH-900475[Title/Abstract])) OR (Keytruda[Title/Abstract])) OR (MK-3475[Title/Abstract])) OR (atezolizumab[Title/Abstract])) OR (MPDL-3280A[Title/Abstract])) OR (RG-7446[Title/Abstract])) OR (durvalumab[Title/Abstract])) OR (MEDI-4736[Title/Abstract])) OR (Imfinzi[Title/Abstract])) OR (avelumab[Title/Abstract])) OR (MSB-0010682[Title/Abstract])) OR (bavencio[Title/Abstract])) OR (MSB-0010718C[Title/Abstract])) OR (Ipilimumab[Title/Abstract])) OR (MDX 010[Title/Abstract])) OR (Yervoy[Title/Abstract])) OR (Tremelimumab[Title/Abstract])) OR (CP-675[Title/Abstract])) OR (camrelizumab[Title/Abstract])) OR (carrelizumab[Title/Abstract])) OR (SHR-1210[Title/Abstract])) OR (cemiplimab[Title/Abstract])) OR (REGN2810[Title/Abstract])) OR (tiragolumab[Title/Abstract])) OR (dostarlimab[Title/Abstract])) OR (GSK4057190[Title/Abstract])) OR (TSR-042[Title/Abstract])) OR (tislelizumab[Title/Abstract])) OR (BGB-A317[Title/Abstract])) OR (sintilimab[Title/Abstract])) OR (IBI 308[Title/Abstract])) OR (toripalimab[Title/Abstract]) | 156,425 |
|  | #5: #3 AND #4 | 361 |
| **Embase** | #1: 'nasopharynx tumor'/exp | 32,670 |
|  | #2:'nasopharyngeal neoplasm':ab,ti OR 'nasopharyngeal neoplasms':ab,ti OR 'neoplasm, nasopharyngeal':ab,ti OR 'neoplasms nasopharyngeal':ab,ti OR 'nasopharynx neoplasms':ab,ti OR 'nasopharynx neoplasm':ab,ti OR 'neoplasm, nasopharynx':ab,ti OR 'neoplasms, nasopharynx':ab,ti OR 'cancer of nasopharynx':ab,ti OR 'nasopharynx cancers':ab,ti OR 'nasopharyngeal cancer':ab,ti OR 'cancer, nasopharyngeal':ab,ti OR 'cancers, nasopharyngeal':ab,ti OR 'nasopharyngeal cancers':ab,ti OR 'nasopharynx cancer':ab,ti OR 'cancer, nasopharynx':ab,ti OR 'cancers, nasopharynx':ab,ti OR 'cancer of the nasopharynx':ab,ti | 3,696 |
|  | #3: #1 OR #2 | 33,042 |
|  | #4: 'pd-1':ab,ti OR 'pd-l1':ab,ti OR 'anti-pd-1':ab,ti OR 'anti-pd-l1':ab,ti OR  'immune checkpoint inhibitor':ab,ti OR 'immune checkpoint inhibitors':ab,ti OR 'ici':ab,ti OR 'icis':ab,ti OR 'immune checkpoint blocker':ab,ti OR 'immune checkpoint blockers':ab,ti OR 'icb':ab,ti OR 'icbs':ab,ti OR 'immunotherapy':ab,ti OR 'ctla-4':ab,ti OR 'anti-ctla-4':ab,ti OR 'nivolumab':ab,ti OR 'opdivo':ab,ti OR 'ono-4538':ab,ti OR 'mdx-1106':ab,ti OR 'bms-936558':ab,ti OR 'pembrolizumab':ab,ti OR 'sch-900475':ab,ti OR  'keytruda':ab,ti OR 'mk-3475':ab,ti OR 'atezolizumab':ab,ti OR 'mpdl-3280a':ab,ti OR 'rg-7446':ab,ti OR 'durvalumab':ab,ti OR 'medi-4736':ab,ti OR 'imfinzi':ab,ti OR 'avelumab':ab,ti OR 'msb-0010682':ab,ti OR 'bavencio':ab,ti OR 'msb-0010718c':ab,ti OR  'ipilimumab':ab,ti OR 'mdx 010':ab,ti OR 'yervoy':ab,ti OR 'tremelimumab':ab,ti OR 'cp-675':ab,ti OR 'camrelizumab':ab,ti OR 'carrelizumab':ab,ti OR 'shr-1210':ab,ti OR 'cemiplimab':ab,ti OR 'regn2810':ab,ti OR 'tiragolumab':ab,ti OR 'dostarlimab':ab,ti OR 'gsk4057190':ab,ti OR 'tsr-042':ab,ti OR 'tislelizumab':ab,ti OR 'bgb-a317':ab,ti OR 'sintilimab':ab,ti OR 'ibi 308':ab,ti OR 'toripalimab':ab,ti | 240,271 |
|  | #5: #3 AND #4 | 854 |
| **Cochrane** | #1: MeSH descriptor: [Nasopharyngeal Neoplasms] explode all trees | 551 |
|  | #2:(Nasopharyngeal Neoplasm):ti,ab,kw OR (Neoplasm, Nasopharyngeal):ti,ab,kw OR (Neoplasms, Nasopharyngeal):ti,ab,kw OR (Nasopharynx Neoplasms):ti,ab,kw OR (Neoplasm, Nasopharynx):ti,ab,kw OR (Neoplasms, Nasopharynx):ti,ab,kw OR (Cancer of Nasopharynx):ti,ab,kw OR (Nasopharynx Cancers):ti,ab,kw OR (Nasopharyngeal Cancer):ti,ab,kw OR (Cancer, Nasopharyngeal):ti,ab,kw OR (Cancers, Nasopharyngeal):ti,ab,kw OR (Nasopharyngeal Cancers):ti,ab,kw OR (Nasopharynx Cancer):ti,ab,kw OR (Cancer, Nasopharynx):ti,ab,kw OR (Cancers, Nasopharynx):ti,ab,kw OR (Cancer of the Nasopharynx):ti,ab,kw | 1304 |
|  | #3: #1 OR #2 | 1359 |
|  | #4:(PD-1):ti,ab,kw OR (PD-L1):ti,ab,kw OR (anti-PD-1):ti,ab,kw OR (anti-PD-1):ti,ab,kw OR (immune checkpoint inhibitor):ti,ab,kw OR (immune checkpoint inhibitors):ti,ab,kw OR (ICI):ti,ab,kw OR (ICIs):ti,ab,kw OR (immune checkpoint blocker):ti,ab,kw OR (immune checkpoint blockers):ti,ab,kw OR (ICB):ti,ab,kw OR (ICBs):ti,ab,kw OR (immunotherapy):ti,ab,kw OR (CTLA-4):ti,ab,kw OR (Anti-CTLA-4):ti,ab,kw OR (Anti-CTLA-4):ti,ab,kw OR (nivolumab):ti,ab,kw OR (Opdivo):ti,ab,kw OR (ONO-4538):ti,ab,kw OR (MDX-1106):ti,ab,kw OR (BMS-936558):ti,ab,kw OR (pembrolizumab):ti,ab,kw OR (SCH-900475):ti,ab,kw OR (Keytruda):ti,ab,kw OR (MK-3475):ti,ab,kw OR (atezolizumab):ti,ab,kw OR (MPDL-3280A):ti,ab,kw OR (RG-7446):ti,ab,kw OR (durvalumab):ti,ab,kw OR (MEDI-4736):ti,ab,kw OR (Imfinzi):ti,ab,kw OR (avelumab):ti,ab,kw OR (MSB-0010682):ti,ab,kw OR (bavencio):ti,ab,kw OR (MSB-0010718C):ti,ab,kw OR (Ipilimumab):ti,ab,kw OR (MDX 010):ti,ab,kw OR (Yervoy):ti,ab,kw OR (Tremelimumab):ti,ab,kw OR (CP-675):ti,ab,kw OR (camrelizumab):ti,ab,kw OR (carrelizumab):ti,ab,kw OR (SHR-1210):ti,ab,kw OR (cemiplimab):ti,ab,kw OR (REGN2810):ti,ab,kw OR (tiragolumab):ti,ab,kw OR (dostarlimab):ti,ab,kw OR (GSK4057190):ti,ab,kw OR (TSR-042):ti,ab,kw OR (tislelizumab):ti,ab,kw OR (BGB-A317):ti,ab,kw OR (sintilimab):ti,ab,kw OR (IBI 308):ti,ab,kw OR (toripalimab):ti,ab,kw | 19,378 |
|  | #5: #3 AND #4 | 102 |
| **Web of Science** | #1: TS=('Nasopharyngeal Neoplasms' OR 'Nasopharyngeal Neoplasm' OR 'Neoplasm, Nasopharyngeal' OR 'Neoplasms, Nasopharyngeal' OR 'Nasopharynx Neoplasms' OR 'Nasopharynx Neoplasm' OR 'Neoplasm, Nasopharynx' OR 'Neoplasms, Nasopharynx' OR 'Cancer of Nasopharynx' OR 'Nasopharynx Cancers' OR 'Nasopharyngeal Cancer' OR 'Cancer, Nasopharyngeal' OR 'Cancers, Nasopharyngeal' OR 'Nasopharyngeal Cancers' OR 'Nasopharynx Cancer' OR 'Cancer, Nasopharynx' OR 'Cancers, Nasopharynx' OR 'Cancer of the Nasopharynx') | 33,995 |
|  | #2:TS=('PD-1' OR 'PD-L1' OR 'anti-PD-1' OR 'anti-PD-L1' OR 'immune checkpoint inhibitOR' OR 'immune checkpoint inhibitORs' OR 'ICI' OR 'ICIs' OR 'immune checkpoint blocker' OR 'immune checkpoint blockers' OR 'ICB' OR 'ICBs' OR 'immunotherapy' OR 'CTLA-4' OR 'Anti-CTLA-4' OR 'nivolumab' OR 'Opdivo' OR 'ONO-4538' OR 'MDX-1106' OR 'BMS-936558' OR 'pembrolizumab' OR 'SCH-900475' OR 'Keytruda' OR 'MK-3475' OR 'atezolizumab' OR 'MPDL-3280A' OR 'RG-7446' OR 'durvalumab' OR 'MEDI-4736' OR 'Imfinzi' OR 'avelumab' OR 'MSB-0010682' OR 'bavencio' OR 'MSB-0010718C' OR 'Ipilimumab' OR 'MDX 010' OR 'Yervoy' OR 'Tremelimumab' OR 'CP-675' OR 'camrelizumab' OR 'carrelizumab' OR 'SHR-1210' OR 'cemiplimab' OR 'REGN2810' OR 'tiragolumab' OR 'dostarlimab' OR 'GSK4057190' OR 'TSR-042' OR 'tislelizumab' OR 'BGB-A317' OR 'sintilimab' OR 'IBI 308' OR 'tORipalimab') | 292,145 |
|  | #3: #1 AND #2 | 1,123 |

**Table S2** Quality assessment of included studies using Newcastle-Ottawa quality assessment scale

| Study | Selection | | | | Comparability | Outcome | | | Total |
| --- | --- | --- | --- | --- | --- | --- | --- | --- | --- |
|  | Representativeness of exposed cohort | Selection of the nonexposed cohort | Ascertainment of exposure to implants | Demonstration that outcome of interest was not present at start of study | Comparability of cohorts based on the design or analysis | Assessment of outcome | Follow-up  long enough  For outcomes  to occur | Adequacy of follow up of cohorts |  |
| Yang 2021[1] (1) | 1 | 1 | 1 | 1 | 2 | 1 | 1 | 1 | 9 |
| Yang 2021[2] (2) | 1 | 1 | 1 | 1 | 2 | 1 | 1 | 1 | 9 |
| Ma 2018(3) | 1 | 1 | 1 | 1 | 2 | 1 | 1 | 1 | 9 |
| Xu J 2022(4) | 1 | 1 | 1 | 1 | 2 | 1 | 1 | 1 | 9 |
| Wang 2021(5) | 1 | 1 | 1 | 1 | 2 | 1 | 1 | 1 | 9 |
| Hua 2021(6) | 1 | 1 | 1 | 1 | 2 | 1 | 1 | 1 | 9 |
| Even 2021(7) | 1 | 1 | 1 | 1 | 2 | 1 | 1 | 1 | 9 |
| Park 2020(8) | 1 | 1 | 1 | 1 | 0 | 1 | 1 | 1 | 7 |
| Fang 2018(9) | 1 | 1 | 1 | 1 | 2 | 1 | 1 | 1 | 9 |
| Mai 2021(10) | 1 | 1 | 1 | 1 | 2 | 1 | 1 | 1 | 9 |
| Ma 2021(11) | 1 | 1 | 1 | 1 | 2 | 1 | 1 | 1 | 9 |
| Xu L 2022(12) | 1 | 1 | 1 | 1 | 2 | 1 | 1 | 0 | 8 |
| Shi 2022(13) | 1 | 1 | 1 | 1 | 2 | 1 | 1 | 1 | 9 |
| Chen 2022(14) | 1 | 1 | 0 | 1 | 2 | 1 | 1 | 1 | 8 |
| Chiang 2022(15) | 1 | 1 | 0 | 1 | 2 | 1 | 1 | 1 | 8 |


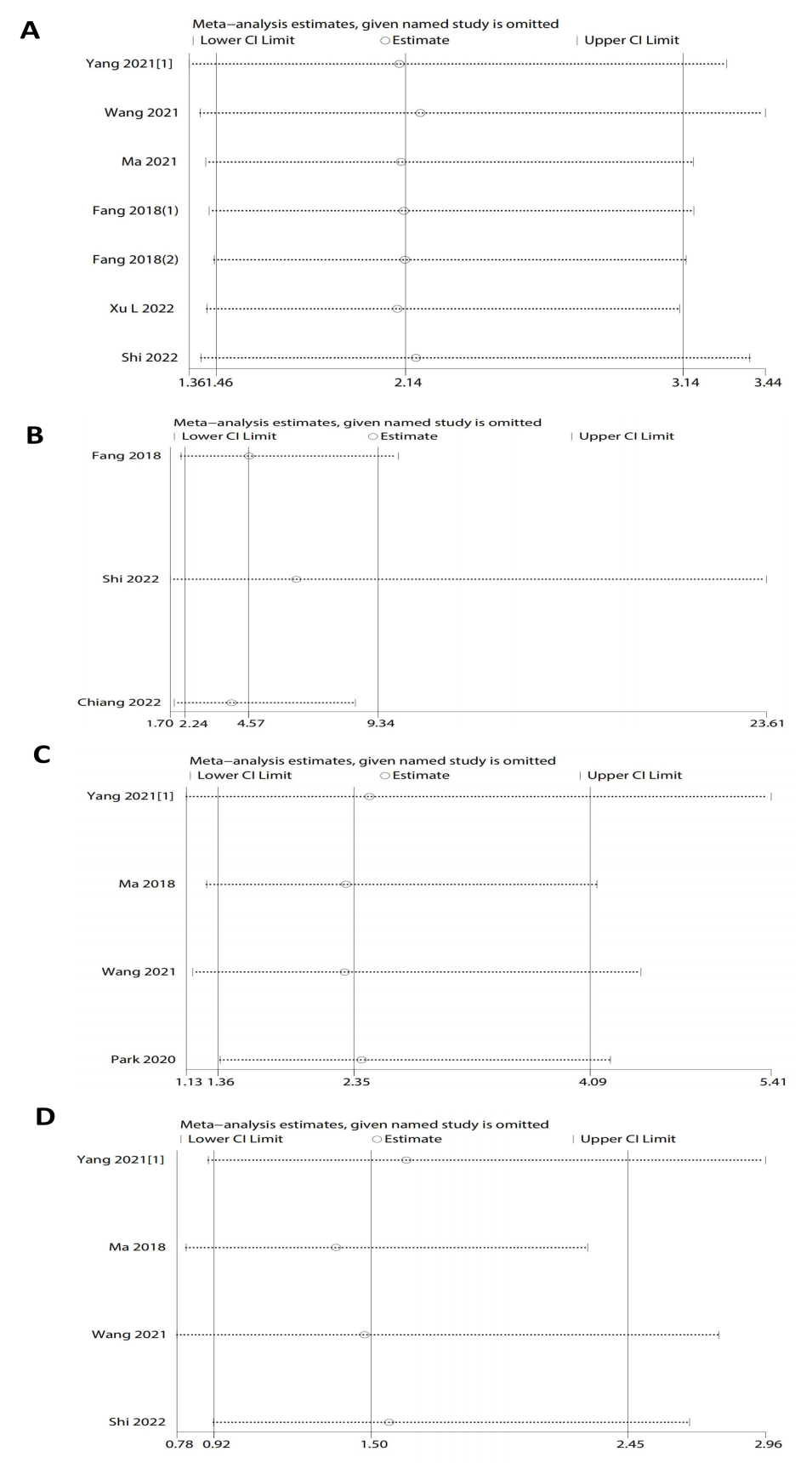


**Figure S1** Sensitivity analysis of studies included in meta-analysis of objective response rate (ORR) for biomarkers. (A) baseline plasma Epstein-Barr virus (EBV) DNA level; (B) dynamic plasma EBV DNA load during immunotherapy; (C) programmed cell death-ligand 1 (PD-L1) expression (higher *vs.* lower); (D) PD-L1 expression (positive *vs.* negative).


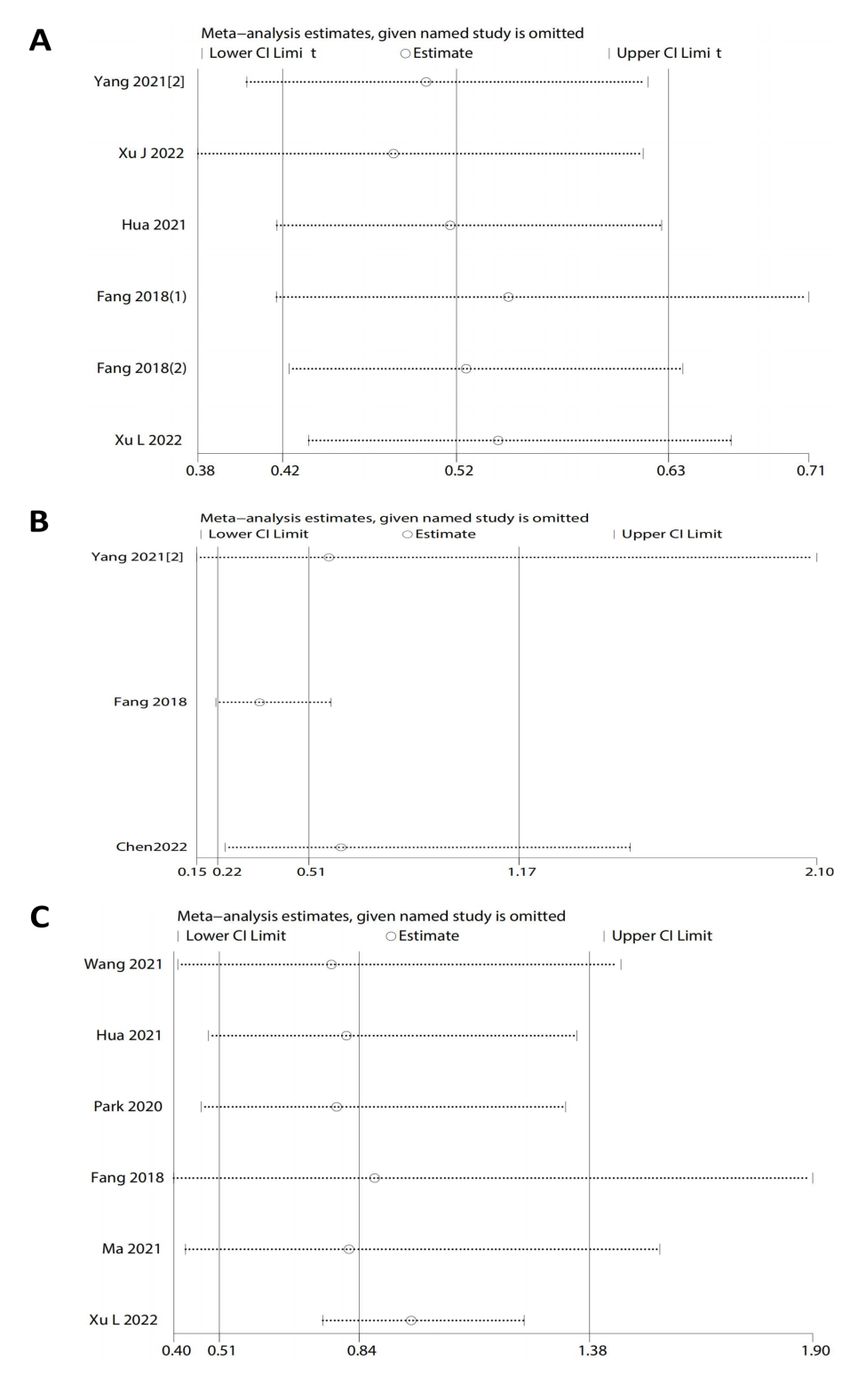


**Figure S2** Sensitivity analysis of studies included in meta-analysis of progression-free survival (PFS) for biomarkers. (A) baseline plasma Epstein-Barr virus (EBV) DNA level; (B) dynamic plasma EBV DNA load during immunotherapy; (C) tumor mutation burden (TMB).

References

1. Yang Y, Zhou T, Chen X, Li J, Pan J, He X, et al. Efficacy, safety, and biomarker analysis of Camrelizumab in Previously Treated Recurrent or Metastatic Nasopharyngeal Carcinoma (CAPTAIN study). *J Immunother Cancer*(2021)9(12). doi: 10.1136/jitc-2021-003790

2. Yang Y, Qu S, Li J, Hu C, Xu M, Li W, et al. Camrelizumab versus placebo in combination with gemcitabine and cisplatin as first-line treatment for recurrent or metastatic nasopharyngeal carcinoma (CAPTAIN-1st): a multicentre, randomised, double-blind, phase 3 trial. *Lancet Oncol*(2021)22(8):1162-74. doi: 10.1016/S1470-2045(21)00302-8

3. Ma BBY, Lim WT, Goh BC, Hui EP, Lo KW, Pettinger A, et al. Antitumor Activity of Nivolumab in Recurrent and Metastatic Nasopharyngeal Carcinoma: An International, Multicenter Study of the Mayo Clinic Phase 2 Consortium (NCI-9742). *J Clin Oncol*(2018)36(14):1412-8. doi: 10.1200/JCO.2017.77.0388

4. Xu JY, Wei XL, Ren C, Zhang Y, Hu YF, Li JY, et al. Association of Plasma Epstein-Barr Virus DNA With Outcomes for Patients With Recurrent or Metastatic Nasopharyngeal Carcinoma Receiving Anti-Programmed Cell Death 1 Immunotherapy. *JAMA Netw Open*(2022)5(3):e220587. doi: 10.1001/jamanetworkopen.2022.0587

5. Wang FH, Wei XL, Feng J, Li Q, Xu N, Hu XC, et al. Efficacy, Safety, and Correlative Biomarkers of Toripalimab in Previously Treated Recurrent or Metastatic Nasopharyngeal Carcinoma: A Phase II Clinical Trial (POLARIS-02). *J Clin Oncol*(2021)39(7):704-12. doi: 10.1200/JCO.20.02712

6. Hua Y, You R, Wang Z, Huang P, Lin M, Ouyang Y, et al. Toripalimab plus intensity-modulated radiotherapy for recurrent nasopharyngeal carcinoma: an open-label single-arm, phase II trial. *J Immunother Cancer*(2021)9(11). doi: 10.1136/jitc-2021-003290

7. Even C, Wang HM, Li SH, Ngan RK, Dechaphunkul A, Zhang L, et al. Phase II, Randomized Study of Spartalizumab (PDR001), an Anti-PD-1 Antibody, versus Chemotherapy in Patients with Recurrent/Metastatic Nasopharyngeal Cancer. *Clin Cancer Res*(2021)27(23):6413-23. doi: 10.1158/1078-0432.CCR-21-0822

8. Park JC, Durbeck J, Boudadi K, Ho WJ, Kang H. The efficacy of anti-PD-1 immune checkpoint inhibitor in nasopharyngeal carcinoma. *Oral Oncol*(2020)108:104935. doi: 10.1016/j.oraloncology.2020.104935

9. Fang W, Yang Y, Ma Y, Hong S, Lin L, He X, et al. Camrelizumab (SHR-1210) alone or in combination with gemcitabine plus cisplatin for nasopharyngeal carcinoma: results from two single-arm, phase 1 trials. *Lancet Oncol*(2018)19(10):1338-50. doi: 10.1016/S1470-2045(18)30495-9

10. Mai HQ, Chen QY, Chen D, Hu C, Yang K, Wen J, et al. Toripalimab or placebo plus chemotherapy as first-line treatment in advanced nasopharyngeal carcinoma: a multicenter randomized phase 3 trial. *Nat Med*(2021)27(9):1536-43. doi: 10.1038/s41591-021-01444-0

11. Ma Y, Chen X, Wang A, Zhao H, Lin Q, Bao H, et al. Copy number loss in granzyme genes confers resistance to immune checkpoint inhibitor in nasopharyngeal carcinoma. *J Immunother Cancer*(2021)9(3). doi: 10.1136/jitc-2020-002014

12. Xu L, Ma Y, Fang C, Peng Z, Gao F, Moll JM, et al. Genomic and microbial factors affect the prognosis of anti-pd-1 immunotherapy in nasopharyngeal carcinoma. *Front Oncol*(2022)12:953884. doi: 10.3389/fonc.2022.953884

13. Shi Y, Qin X, Peng X, Zeng A, Li J, Chen C, et al. Efficacy and safety of KL-A167 in previously treated recurrent or metastatic nasopharyngeal carcinoma: A multicenter, single-arm, phase 2 study. *The Lancet Regional Health – Western Pacific*. doi: 10.1016/j.lanwpc.2022.100617

14. Chen SY, Chen M, Rui Y, Hua Y, Zou X, Wang ZQ. Efficacy and safety of chemotherapy plus subsequent locoregional radiotherapy and toripalimab in de novo metastatic nasopharyngeal carcinoma. *Journal of Clinical Oncology*(2022)40(16). doi: 10.1200/JCO.2022.40.16_suppl.6025

15. Chiang CL, Lam TC, Li CBJ, Li WS, Chan SK, Lee YPY, et al. Antitumor activity of bintrafusp alfa in previously treated patients with recurrent or metastatic nasopharyngeal cancer (NPC): A single arm, prospective phase II trial. *Journal of Clinical Oncology*(2022)40(16). doi: 10.1200/JCO.2022.40.16_suppl.e18029
